# Distance to radiation therapy facility influences surgery type among older women with early‐stage breast cancer

**DOI:** 10.1002/cam4.5474

**Published:** 2022-12-09

**Authors:** Pratibha Shrestha, Quyen D. Chu, Mei‐Chin Hsieh, Yong Yi, Edward S. Peters, Edward Trapido, Qingzhao Yu, Tekeda Ferguson, Xiao‐Cheng Wu

**Affiliations:** ^1^ Louisiana Tumor Registry, Epidemiology Program, School of Public Health at LSU Health Sciences Center‐New Orleans New Orleans Louisiana USA; ^2^ Orlando Health Cancer Institute Orlando Florida USA; ^3^ Department of Epidemiology, UNMC College of Public Health Omaha Nebraska USA; ^4^ Biostatistics Program, School of Public Health at LSU Health Sciences Center‐New Orleans New Orleans Louisiana USA

**Keywords:** breast‐conserving therapy, distance to radiation facility, early‐stage breast cancer, mastectomy

## Abstract

**Background:**

Breast‐conserving surgery plus radiation (BCT) yields equivalent or better survival than mastectomy for early‐stage breast cancer (ESBC) women. However, nationwide mastectomy trends increased in recent decades, attracting studies on underlying causes. Prior research identified that long distance to the radiation treatment facility (RTF) was associated with mastectomy. Still, it is unclear whether such association applies to young and old ESBC women comparably. We sought to delineate such impacts by age.

**Methods:**

Women diagnosed with stages 0–II breast cancer in 2013–2017 receiving either BCT or mastectomy were identified from the Louisiana Tumor Registry. We assessed the association of surgery (mastectomy vs. BCT) with the distance to the nearest or nearest accessible RTFs using multivariable logistic regression adjusting the socio‐demographic and tumor characteristics. The nearest accessible RTF was determined based on patients' health insurance. For Medicaid, uninsured, and unknown insurance patients, the nearest accessible RTF is the nearest RTF owned by the government. The interaction effect of age and distance was evaluated as well.

**Results:**

Of 11,604 patients, 46.7% received mastectomy. Compared with distance ≤5 miles to the nearest RTF, those with distance ≥40 miles or 15–40 miles had higher odds of mastectomy (adjusted (adj) OR = 1.39, 95% CI = 1.07–1.82; adj OR = 1.17, 95% CI = 1.02–1.34). To the nearest accessible RTF, the adj ORs were 1.25 (95% CI = 1.03–1.51) and 1.19 (95% CI = 1.04–1.35), respectively. Age‐stratified analysis showed the significant association (*p* < 0.05) only presented among women aged ≥65, but not those aged <65 years.

**Conclusion:**

Distance to the nearest or nearest accessible RTF influences the surgery choice, especially among women in Louisiana ≥65 years with ESBC. Further understanding of factors leading to the decision for mastectomy in this age group is needed.

## Graphical abstract

Distance to the radiation therapy facility significantly affects the odds of mastectomy for older women (age = 65 years), but not younger women eligible for breast‐conserving surgery plus radiation. Target interventions that assist older women in overcoming barriers to RTFs may increase the rate of breast‐conserving surgery.

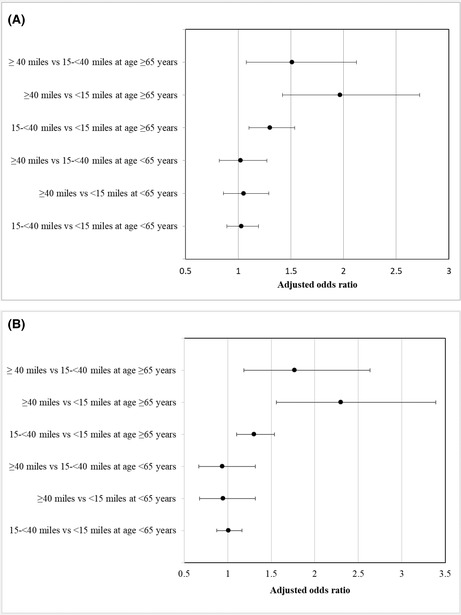

## 1 INTRODUCTION

Breast conserving surgery plus radiation (BCT) has been the treatment for most women with early‐stage breast cancer (ESBC) after the panel recommendation of the 1990 National Institutes of Health (NIH) consensus conference.^1^ The recommendation was based on the randomized trials conducted in the 1980s that illustrated equivalent survival of BCT as mastectomy.^2^, ^3^, ^4^ Later, multiple randomized clinical trials also demonstrated that BCT has equivalent overall survival as mastectomy,^5^, ^6^, ^7^, ^8^ a short recovery period,^9^ and less severe psychosocial stress regarding body image after surgery.^10^ A study conducted among ESBC women diagnosed in 1990–2004 observed that women undergoing BCT exhibited better survival than those who received mastectomy.^11^ In addition, recent publications^12^, ^13^, ^14^, ^15^, ^16^, ^17^ reported that BCT results in improved survival compared to mastectomy alone for ESBC patients. Despite these documented advantages of BCT, mastectomy for ESBC increased nationwide over the past two decades, prompting interest to study the rationale behind these trends.^18^, ^19^, ^20^

Factors associated with the increased receipt of mastectomy include large tumor size, patients residing in rural areas, low body mass index, younger age, fear of cancer recurrence, frequent visit requirements, and long distance to radiation treatment facility (RTF).^21^, ^22^, ^23^, ^24^ To receive post‐surgical radiation therapy, a woman usually has to commute between their residence and the radiation facility every day for 3–6 weeks after breast‐conserving surgery, which may be difficult, especially for older women without available transportation. Distance to RTF is one of the most influential nonclinical factors in receiving mastectomy.^23^ Besides the distance to RTF, the age of the women might influence surgery choices^25^ as younger women are more likely to elect to undergo mastectomy for an aggressive breast cancer^26^; yet, chose BCT to preserve the breast with less aggressive cancers. However, older women may elect mastectomy regardless of aggressiveness, as they are more likely to have driving constraints compared to younger women.^27^ Thus, age may have a moderate relation with distance to RTF in receipt of surgery treatment. A national cohort study observed that the women residing 15 miles or more from the nearest hospital with a radiation facility had a 48% lower probability of undergoing a lumpectomy.^28^ Also, those who underwent lumpectomy had a lower likelihood of receiving radiotherapy. This study also examined the age and distance association with surgery, but the analysis is limited to older women only, with limited covariates adjustment among those who underwent BCT.^28^ Acharya et al. reported that the odds of receiving mastectomy versus BCT increased with the increasing travel distance to RTF.^23^ Similarly, a study done in Virginia showed that women with ESBC receiving mastectomy increased with distance to RTF (43% at ≤10 miles, 47% at 10–25 miles, 53% at 25–50 miles, and 58% at >50 miles, *p* < 0.001).^24^ A recent study conducted among Medicare recipients (2004–2013) in women aged ≥65 years showed that women living farther from RTF were more likely to undergo mastectomy versus lumpectomy. Among women who underwent a lumpectomy, those who live farther from RTF were less likely to receive radiation therapy.^25^ However, this study^25^ limited the investigation only to women aged ≥65 years enrolled in Medicare, limiting the study's generalization to all aged women and not adjusting for HER2 and progesterone status. Other prior studies^19^, ^23^ compared BCT and mastectomy, adjusted for age but did not examine how age can partially moderate the association of distance to RTF with treatment choices.

Most studies did not delineate whether the nearest RTF is also one that is accessible to the patients. Since not all RTF accept Medicaid/uninsured patients, these patients may have to travel a farther distance to an accessible RTF. Our study specifically examined the distance to the nearest RTFs and the nearest accessible RTFs; the latter are government‐owned RTFs accessible to Medicaid/no insurance patients. This study aimed to assess (1) the impact of the distance to the nearest or the nearest accessible RTF on surgery choice and (2) the differences in the above impact between young and old women.

## 2 METHODS AND MATERIALS

### 2.1 Data source and study population

Data were from the population‐based Louisiana Tumor Registry (LTR).^29^ The eligible patients were Louisiana women diagnosed with American Joint Committee on Cancer (AJCC) stage 0‐II breast cancer (BC) in 2013–2017^30^ and received either BCT or mastectomy. Unknown race (*n* = 2), unknown poverty or urbanization (*n* = 3), and outlier body weight or height (*n* = 17) were excluded. This research was reviewed and approved by the Louisiana State University Health Science Centers Institutional Review Board IRB #2368.

Information on the government‐owned RTFs (accessible to all patients) and private RTFs were from the American College of Surgeons (ACoS) and Association of Health Care Journalists (AHCJ) websites^31^, ^32^ verified with the local RTF offices if the information was not available on any of the websites.

### 2.2 Outcome

The surgery type was the outcome variable with two groups: mastectomy (site‐specific surgery codes 30–80) and BCT (site‐specific surgery codes 19–24 plus radiation therapy).^33^

### 2.3 Exposure: Distance to RTF

The primary interest of the exposure was the distance to RTF, which was classified as the nearest RTF and nearest accessible RTF/government‐owned. We identified 42 Louisiana RTFs treating BC patients from the LTR database. The distances from patients' residential addresses to the nearest government‐owned RTFs, and private RTFs in Louisiana were calculated using the Haversine formula,^34^ which calculates the shortest great‐circle distance between two points on a sphere from their longitudes and latitudes.

The nearest RTF is closest to patients' homes, regardless of whether owned by the government or private. The nearest accessible RTF was determined based on patients' health insurance. For Medicaid, uninsured, and unknown insurance patients, the nearest accessible RTF is the nearest RTF owned by the government, which provided more precise distance estimates for these patients because some private RTF do not accept Medicaid/uninsured patients. While, for patients with Medicare, Private, and government support insurance (Tricare, Military, Veterans Affairs, and Indian/ Public Health Service), the nearest accessible RTF is any nearest RTF. The distance measured by miles was categorized as ≤5, >5 to <15, ≥15 to <40, and ≥ 40.^23^

### 2.4 Covariates

Covariates included age at diagnosis (<45, 45–54, 55–64, 65–74, and ≥ 75 years), race (White, Black, and Others), body mass index (which was calculated in kg/m^2^ and classified as underweight <18.5, normal weight 18.5‐ < 25, overweight 25‐ < 30, obesity ≥30, and unknown),^35^ diagnosis year, poverty at the residential census tract under the federal poverty level (0%–<10%, 10‐ < 20%, and ≥ 20%) and urbanity represents the percent of the population in an urban area at census tract level (all urban 100%, mostly urban ≥50%–<100%, mostly rural >0 ‐ < 50%, and all rural 0%),^36^ insurance status at diagnosis (not insured, Medicaid, Private, Medicare, government support insurance including Tricare, Military, Veterans Affairs, and Indian/ Public Health Service, and unknown),^37^ and Charlson/Deyo Comorbidity Scores (CCS: based on 16 comorbidities and classified as 0, 1, and 2+ comorbidity scores).^38^

For interaction analysis, age was re‐categorized into women aged <65 years (young) and aged ≥65 years (old).^27^, ^28^

Specific cancer‐related covariates were AJCC stages (0, I, II), and subtype of hormone receptors (HR) and human epidermal growth factor 2 (HER2) were coded as HR+/HER2+, HR+/HER2−, HR−/HER2+, and HR−/HER2−. The hormone receptor (HR) status for BC was based on the test results of estrogen receptor (ER) and progesterone receptor (PR), and they were classified as HR+ (ER+ and/or PR+) or HR− (ER− and PR−).^39^ The borderline ER or PR were grouped with ER+ or PR+. However, for HER2, the positive includes only the positive test. The borderline result was included in the negative group.^40^, ^41^

### 2.5 Statistical analysis

Descriptive analysis and Chi‐square tests were performed by type of surgery received, respectively. The significance level for *p*‐values was set at 0.05. Univariate logistic regression was carried out to assess the individual association of covariates with the ESBC surgery received. Multivariable logistic regression was used to examine the association of ESBC surgery type received with the distance to the nearest RTF (Model 1) and the nearest accessible RTF (Model 2), respectively. Unadjusted and adjusted odds ratios (ORs) and 95% confidence intervals (CIs) were calculated. The interaction term was created by re‐categorize age groups to young (age < 65 years) and old (age ≥ 65 years) and distance by <15 miles, 15‐ < 40 miles, and ≥ 40 miles. All data analyses were performed using SAS 9.4.

## 3 RESULTS

Of 11,604 women with early stages (0‐II) BC, almost two‐fifths of the patients were age 65 and older (38.5%); the median age was 61 years (Table 1). The majority of patients were white (69%), privately insured (51.6%), resided in urban areas (77.2%), overweight/obese (67.9%), and without Charlson comorbidity (81.3%). Patients with HR+/HER2‐breast cancer accounted for 58.9% of the cases, and triple‐negative breast cancer (TNBC) for 11.6%. Almost half of the patients resided within ≤5 miles from the nearest RTF (49.6%) and nearest accessible RTF (46.7%).

**TABLE 1:** Early‐stage (0‐II) breast cancer patients and tumor characteristics in Louisiana, 2013–2017

Variables	Frequency (*N* = 11,604)	Percentage (%)
Age at diagnosis (year)		
< 45	1250	10.8
45–54	2412	20.8
55–64	3465	29.9
65–74	3020	26.0
75+	1457	12.5
Race		
White	8002	69.0
Black	3427	29.5
Others	175	1.5
Diagnosis year		
2013	2214	19.1
2014	2254	19.4
2015	2395	20.6
2016	2384	20.5
2017	2357	20.3
Census tract poverty %^a^		
<10	3261	28.1
10‐ < 20	4064	35.0
20‐ < 100	4279	36.9
Insurance		
Not insured	339	2.9
Medicaid	889	7.7
Private insurance	5990	51.6
Medicare	4095	35.3
Government support insurance^b^	163	1.4
Unknown	128	1.1
Urbanity^c^		
All urban (100%)	5382	46.4
Mostly urban (≥ 50‐ < 100%)	3571	30.8
Mostly rural (0%–50%)	1600	13.8
All rural (0%)	1051	9.0
Body mass index (BMI) in kg/m^2^		
Underweight (<18.5)	115	1.0
Normal weight (18.5‐ < 25)	2377	20.5
Overweight (25‐ < 30)	3019	26.0
Obesity (≥30)	4867	41.9
Unknown	1226	10.6
AJCC stage		
0	2086	18.0
I	5630	48.5
II	3888	33.5
Subtype of HR/HER2		
HR+/HER2+	963	8.3
HR+/HER2−	6840	58.9
HR−/HER2+	460	4.0
HR−/HER2− (Triple‐negative)	1341	11.6
Unknown	2000	17.2
Charlson comorbidity score		
0	9437	81.3
1	1629	14.0
2+	538	4.6
Distance (mile) to the nearest RTF^d^		
≤5	5751	49.6
>5‐ < 15	3506	30.2
15‐ < 40	2058	17.7
≥ 40	289	2.5
Distance (mile) to the nearest accessible RTF^d^		
≤5	5416	46.7
>5‐ <15	3332	28.7
15‐ < 40	2016	17.4
≥ 40	840	7.2

Abbreviations: AJCC, American Joint Committee on Cancer; HR/ HER2, hormone receptors/human epidermal growth factor 2–neu; RTF, radiation therapy facilities.

^a^ Census tract poverty level indicates the percent of inhabitants living below the poverty threshold in a given census tract.

^b^ Government support insurance includes Tricare, Military, Veterans Affairs, and Indian/ Public Health Service.

^c^ Urbanity represents the percentage of population distribution in urban areas.

^d^ Distance (mile) to RTF was measured by using great circle distance.

Overall, 46.7% of the patients received mastectomy (Table 2). In the unadjusted model, younger age, high poverty area, Medicaid insured, all rural area, underweight, stage II, HR‐/HER2+ tumor, and comorbidity score 2+ predicted higher odds of mastectomy than their corresponding counterparts. Compared with distance ≤5 miles to the nearest RTF, higher odds of mastectomy was observed for the distance >15–<40 miles and ≥ 40 miles (OR = 1.20, 95% CI = 1.09–1.33; OR = 1.56, 95% CI = 1.23–1.97, respectively), but not for the distance >5–<15 miles. Similarly, those with a distance >15–<40 miles or ≥ 40 miles to the nearest accessible RTF also had higher odds of mastectomy than those with a distance ≤5 miles (OR = 1.22, 95% CI = 1.10–1.35 and OR = 1.48, 95% CI = 1.28–1.71, respectively), but not for the distance >5–<15 miles.

**TABLE 2:** Association of demographic and clinical variables with surgery type for early‐stage (0‐II) breast cancer cases diagnosed in Louisiana, 2013–2017

Variables	Mastectomy (*n* = 5414, 46.7%)		BCT (*n* = 6190, 53.3%)		Univariate model	
	*n*	%	*n*	%	OR	95% CI
Age at diagnosis (year)^a^						
<45	839	67.1	411	32.9	1.00	
45–54	1233	58.1	1179	48.9	**0.51**	**0.44**–**0.59**
55–64	1441	41.6	2024	58.4	**0.35**	**0.30**–**0.40**
65–74	1183	39.3	1832	60.7	**0.32**	**0.28**–**0.37**
75+	713	48.9	744	51.1	**0.47**	**0.40**–**0.55**
Race						
White	3752	46.9	4250	53.1	1.00	
Black	1576	46.0	1851	54.0	0.96	0.90–1.00
Others	86	49.1	89	50.9	1.10	0.80–1.50
Diagnosis year^a^						
2013	1125	50.8	1089	49.2	1.00	
2014	1054	46.8	1200	53.2	**0.85**	**0.76**–**0.96**
2015	1091	45.6	1304	54.4	**0.81**	**0.72**–**0.91**
2016	1100	46.1	1284	53.9	**0.83**	**0.74**–**0.93**
2017	1044	44.3	1313	55.7	**0.77**	**0.69**–**0.87**
Census tract poverty %^b^						
0 < 10	1469	45.1	1792	54.9	1.00	
10–<20	1915	47.1	2149	52.9	1.09	0.99–1.19
20 < 100	2030	47.4	2249	52.6	**1.10**	**1.01**–**1.20**
Insurance^a^						
Not insured	173	51.0	166	49.0	1.15	0.93–1.43
Medicaid	453	51.0	436	49.0	1.15	0.99–1.32
Private	2846	47.5	3144	52.5	1.00	
Medicare	1800	44.0	2295	56.0	**0.87**	**0.80**–**0.94**
Government support insurance^a^ ^,^ ^c^	74	45.4	89	54.6	0.92	0.67–1.26
Unknown	68	53.1	60	46.9	1.25	0.88–1.79
Urbanity^a^ ^,^ ^d^						
All urban (100%)	2434	45.2	2948	54.8	**0.78**	**0.69**–**0.90**
Mostly urban (≥50–<100%)	1682	47.1	1889	52.9	**0.85**	**0.74**–**0.97**
Mostly rural (0‐50%)	759	47.4	841	52.6	0.86	0.73–1.00
All rural (0%)	539	51.3	512	48.7	1.00	
Body mass index (BMI) in kg/m^2^ ^a^						
Underweight (<18.5 BMI)	73	63.5	42	36.5	**1.50**	**1.02**–**2.21**
Normal Weight (18.5–<25 BMI)	1277	53.7	1100	46.3	1.00	
Overweight (25–<30 BMI)	1367	45.3	1652	54.7	**0.71**	**0.64**–**0.79**
Obesity (≥30 BMI)	2089	42.9	2778	57.1	**0.65**	**0.59**–**0.72**
Unknown	608	49.6	618	50.4	**0.85**	**0.74**–**0.97**
AJCC stage^a^						
0	849	40.7	1237	59.3	**0.43**	**0.38**–**0.48**
I	2171	38.6	3459	61.4	**0.39**	**0.36**–**0.43**
II	2394	61.6	1494	38.4	1.00	
Subtype of HR/HER2^a^						
HR+/HER2+	501	52.0	462	48.0	1.00	
HR+/HER2−	3087	45.1	3753	54.9	0.76	0.66–0.87
HR−/HER2+	272	59.1	188	40.9	**1.33**	**1.07**–**1.67**
HR−/HER2− (Triple‐negative)	702	52.4	639	47.6	1.01	0.86–1.20
Unknown	852	42.6	1148	57.4	**0.68**	**0.59**–**0.80**
Charlson comorbidity score						
0	4393	46.5	5044	53.5	1.00	
1	745	45.7	884	54.3	0.97	0.87–1.08
2+	276	51.3	262	48.7	**1.21**	**1.02**–**1.44**
Distance (miles) to the nearest RTF^a^ ^,^ ^e^						
≤5	2592	45.1	3159	54.9	1.00	
>5–<15	1639	46.7	1867	53.3	1.07	0.98–1.16
15–<40	1021	49.6	1037	50.4	**1.20**	**1.09**–**1.33**
≥40	162	56.1	127	43.9	**1.56**	**1.23**–**1.97**
Distance (miles) to the nearest accessible RTF^a^ ^,^ ^e^						
≤5	2411	44.5	3005	55.5	1.00	
>5– <15	1549	46.5	1783	53.5	1.08	0.99–1.18
15–<40	998	49.5	1018	50.5	**1.22**	**1.10**–**1.35**
≥40	456	54.3	384	45.7	**1.48**	**1.28**–**1.71**

Abbreviations: AJCC, American Joint Committee on Cancer; BCT, breast‐conserving surgery plus radiation therapy, HR/HER2, hormone receptors/human epidermal growth factor 2 –neu, OR, odds ratio, RTF, radiation therapy facilities. Bold indicates statistically significant values.

^a^ *p*‐value <0.05 for chi‐square test of treatment received and each variable.

^b^ Census tract poverty level indicates the percent of inhabitants living below the poverty threshold in a given census tract.

^c^ Government support insurance includes Tricare, Military, Veterans Affairs, and Indian/Public Health Service.

^d^ Urbanity represents the percentage of population distribution in urban areas.

^e^ Distance to RTF was measured by using great circle distance.

### 3.1 Adjusted models without interaction (Model 1 and Model 2)

After adjusting for covariates, the odds of mastectomy were higher for patients traveling 15–40 miles versus ≤ 5 miles to the nearest RTF (OR = 1.17, 95% CI = 1.02–1.34) and the nearest accessible RTF (OR = 1.19, 95% CI = 1.04–1.36) (Table 3). Patients with distance ≥40 miles to the nearest RTF had 39% higher odds of mastectomy than those with ≤5 miles (OR = 1.39, 95% CI = 1.06–1.82). Similarly, those with ≥40 miles distance to the nearest accessible RTF had 25% higher odds of mastectomy than those with ≤5 miles (OR = 1.25, 95% CI = 1.03–1.51). However, compared with patients with ≤5 miles, the odds of mastectomy were not significantly higher for those with >5–<15 miles to the nearest RTF (OR = 1.06, 95% CI = 0.96–1.18) and the nearest accessible RTF (OR = 1.07, 95% CI = 0.96–1.19).

**TABLE 3:** Association of distance to RTF with surgery type adjusted for demographic and clinical variables, early‐stage (0‐II) breast cancer cases diagnosed in Louisiana, 2013–2017

Odds ratio for received treatment mastectomy vs. BCT				
Variable	Adjusted OR		Adjusted OR	
	Nearest RTF (Model 1)^a^		Nearest accessible RTF (Model 2)^a^	
	OR	95% CI	OR	95% CI
**Distance (miles) to RTF**				
≤5	1.00		1.00	
>5–<15	1.06	0.96–1.18	1.07	0.96–1.19
15–<40	**1.17**	**1.02**–**1.34**	**1.19**	**1.04**–**1.36**
≥40 miles	**1.39**	**1.06**–**1.82**	**1.25**	**1.03**–**1.51**

Abbreviations: BCT, breast‐conserving surgery plus radiation therapy; CI, confidence interval; OR, odds ratio; RTF, radiation therapy facilities. Bold indicates statistically significant values.

^a^ In both models 1 and 2, the age at diagnosis, race, diagnosis year, census tract poverty indicator, insurance, urbanity, BMI, AJCC stage, subtype of HR/HER2, and Charlson comorbidity score were adjusted.

### 3.2 Adjusted models with distance‐age interaction

**FIGURE 1:**
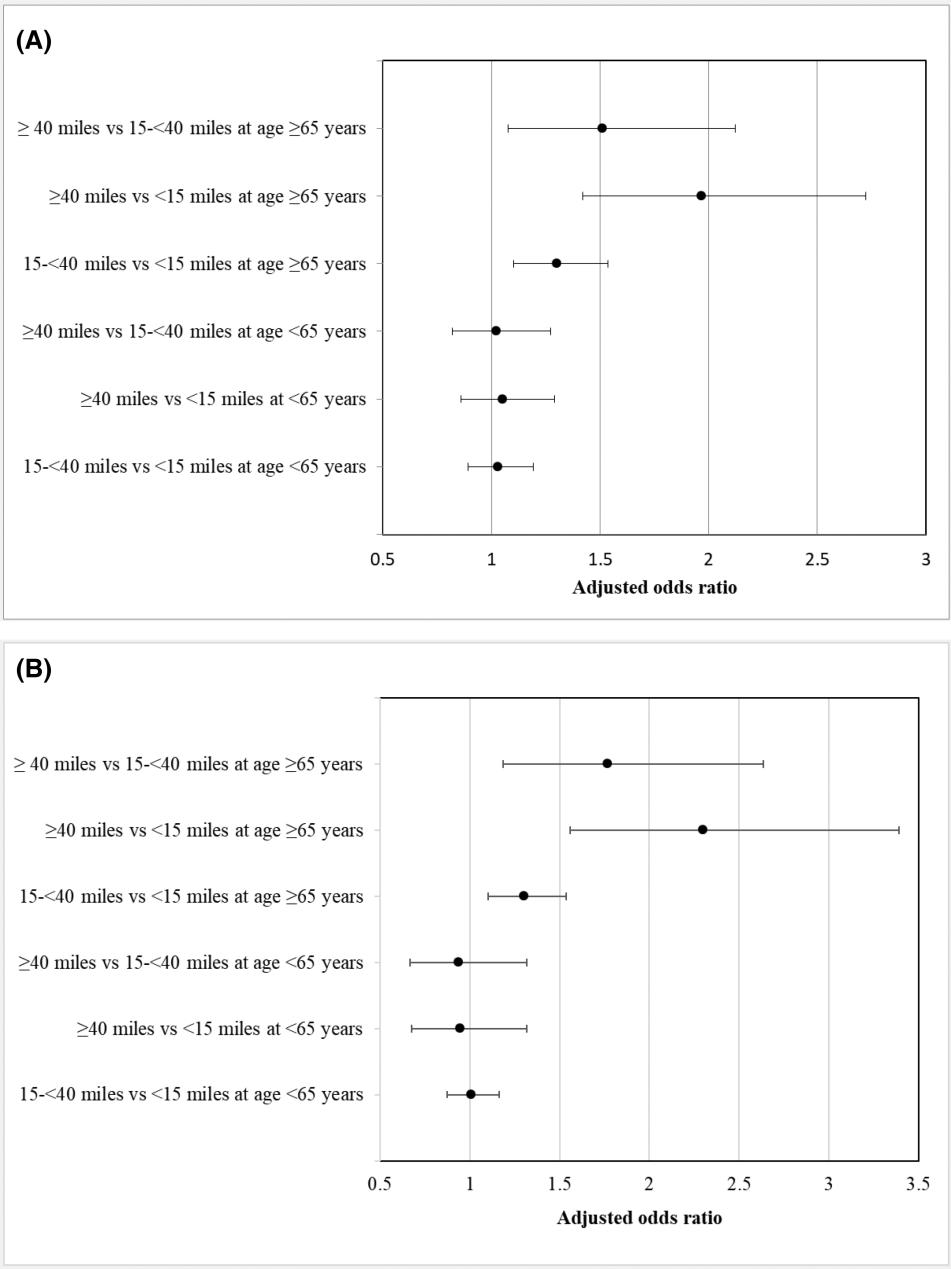
(A) Distance to the nearest accessible RTF. (B) Distance to the nearest RTF.

For patients aged ≥65 years, compared with distance <15 miles or 15‐ < 40 miles to the nearest accessible RTF, distance ≥40 miles had 97% and 51% higher odds of mastectomy, respectively (OR = 1.97, 95% CI = 1.42–2.72 and OR = 1.51, 95% CI = 1.08–2.12) after adjusting for covariates (Figure 1A). To the nearest RTF, the corresponding ORs were 2.30 (95% CI = 1.56–3.39) and 1.77 (95% CI = 1.19–2.64) for patients aged ≥65 years (Figure 1B). In contrast, for patients aged <65 years, the association of the distance to the nearest or nearest accessible RTF was not statistically significant (Figure 1).

## 4 DISCUSSION

We found the effect of age in the association of distance to nearest RTF/nearest accessible RTF and surgery choices in our interaction model, in which we re‐categorized the age group into <65 years (younger women) and ≥ 65 years (older women). For the first time, we found that the distance to the nearest accessible RTF and distance to the nearest RTF affect the odds of mastectomy only among women aged 65 years and older but not among those under age 65. The odds of receiving mastectomy versus BCT were significantly higher among older women aged ≥65 years who resided ≥15 miles from the nearest or nearest accessible RTF. We did not find another study that can be exactly compared to our study results from the interaction model. Still, a cohort study conducted among BC women diagnosed in 1991–1992 found that the odds of receiving radiotherapy among women who did lumpectomy were significantly lower (ORs = 0.56, 95% CI = 0.32–0.97) among the older women aged ≥65 years who resided in ≥40 miles versus <10 miles distance from the hospital with radiation facility.^28^ Therefore, our study‐comparing mastectomy versus BCT provides more clarity about the moderation effect of age (young and older women) and distance to RFT for the surgery choices.

Older women were more likely to have constraints on driving ability, suffer from chronic illnesses, and live in rural areas than younger women.^27^ This may suggest that older patients do not prefer to travel or drive a longer distance to RTF, especially if this requires multiple trips, but opted for mastectomy. However, for younger women, distance to RTFs does not appear to affect their surgical choices. Younger women who opted for mastectomy may be primarily concerned with the risk for loco‐regional recurrence (LRR) and aggressive tumors^26^ that are more likely to grow after undergoing BCT than in older women.^42^ Thus, interventions to help older women overcome the transportation barrier regarding their surgery choices may increase BCT rate.

The other significant finding from our study is that the farther distance to the nearest RTF and the nearest accessible RTF predicts the higher odds of mastectomy; prior studies have not reported the association of the distance to the nearest accessible RTF with the mastectomy. In both adjusted models without interaction, with the increase in distance to nearest RTF or nearest accessible RTF, the odds of receiving mastectomy versus BCT had increased statistically significant. Women who resided 15‐ < 40 miles versus ≤5 miles distance to nearest RTF had 17% higher odds of mastectomy and women with distance 15‐ <40 miles versus ≤5 miles distance to the nearest accessible RTF had 19% higher odds of mastectomy. Similarly, Acharya et al. study found that the women residing within 15‐ < 40 miles versus ≤5 miles to the nearest RTF had 20% (OR = 1.19, 95% CI = 1.05–1.35) higher odds of receiving mastectomy versus BCT.^23^ Likewise, the study conducted among BC women diagnosed in 2004–2006 using 10 US state cancer registries data found that women who need to travel 60‐ < 75 km versus <15 km distance to the nearest RTF had 33% (OR = 1.33, 95% CI = 1.18–1.50) higher odds of receiving mastectomy than BCT.^43^ Other studies also showed that the travel distance or time to RTF was the significant independent predictor for choosing the mastectomy.^44^, ^45^ Thus, it is evident that the transportation barrier is a valid concern for getting optimum breast cancer treatment.

BCT has been considered appropriate alternative for mastectomy for ESBC women or vice versa if patients are unable to receive radiation. However, recent studies^12^, ^13^, ^14^, ^15^, ^16^, ^17^ found that women receiving BCT had better survival than women receiving mastectomy. Especially in our earlier study, we found that the hazard of all‐cause death and breast cancer‐specific deaths in Louisiana (2004–2016) were significantly higher (28.6% and 29.8%) in the mastectomy group versus BCT group.^16^ This implies to prefer BCT instead of mastectomy whenever possible. Thus, to increase the proportion of receiving BCT among ESBC women, our study indicates that older women may need help with transportation. A systematic review study finds that provision of bus passes, taxi vouchers, and reimbursement for transportation may overcome the distance barrier.^46^ For instance, the follow‐up for abnormal Pap smears screening had increased significantly with transportation incentives in clinical trials, especially among the subgroup of women who were more disadvantaged socioeconomically and were at higher risk of cervical cancer.^47^ In contrast in Indiana, a change in policy requiring prior authorization for transportation reimbursement through Medicaid reported a significant decline in visits for medication refills.^48^ Thus, identifying the vulnerable group and the appropriate intervention to overcome distance is important.

Additionally, there can be alternative ways to provide optimal treatment to older patients with ESBC except for addressing the transportation barrier. The traditional way of delivering standard whole breast irradiation (WBI) over 5–7 weeks after breast‐conserving surgery can be replaced by hypo‐fractionated regimes such as five fraction WBI or partial breast irradiation, which can reduce the duration of treatment and the volume of tissue irradiated.^49^ The FAST‐ Forward trial conducted in the UK found that 26 Gray (Gy) in five fractions over a week is not inferior to the standard 40 Gy in 15 fractions over 3 weeks for ESBC patients after primary surgery to control the local tumor, and prevent tissue effects up to 5 years.^50^ Among older women aged ≥70 years diagnosed with clinical stage I and estrogen‐positive breast cancer treated by lumpectomy, Cancer and Leukemia Group B (CALGB) 9343 randomized trial found there were no significant differences in outcomes (overall survival, breast cancer‐specific survival, and time to distant metastasis) among those who received tamoxifen plus radiation therapy or tamoxifen only.^51^ Similarly, the Prime II trial study found that for women aged ≥65 years or older with early hormone receptor‐positive, node‐ negative BC after breast‐conserving surgery, adjuvant endocrine treatment alone can be a reasonable therapeutic treatment for some women. However, this Prime II trial study lacks information on comorbidities.^52^ Thus, we recognize that there may be other treatment options such as hormonal therapy for estrogen‐positive healthy older women, after careful consideration of other factors such as comorbidities. Future studies might address transportation interventions, shorter regimens, and omitting radiation for older women to increase the choices for optimal BC treatment.

We did not find significant differences in the association of the distance with the odds of mastectomy between the nearest RTFs and nearest accessible RTFs. It may be partially attributable to the small proportion of Medicaid/uninsured/unknown insurance BC patients in our study. Although Louisiana has the highest proportion of Medicaid insured population (24.1%–36.3%)^53^ and the average proportion of the uninsured population (8.9%)^54^ compared to other states. Thus, it may be appropriate to examine the association of surgery type with the distance to the nearest RTF, not to the nearest accessible RTF if the proportion of Medicaid/ uninsured patients is low.

The strength of our study is that it is a population‐based study with detailed patient and tumor characteristics. Distance to the nearest accessible RTF was used to delineate the association with the surgery received for women with ESBC in Louisiana. The distances to the nearest and nearest accessible RTF were calculated based on facility and residential addresses, not based on zip code central points, therefore, they are more accurate. The interaction effect of age and distance regarding the surgery received (mastectomy or BCT) was one of the unique findings of this study, which would help health care professionals or policymakers to develop effective interventions to achieve quality care for old ESBC patients.

Our study has a few limitations. Since not all‐private RTF accepted Medicaid or uninsured patients, we only considered government‐owned RTF accessible by these patients, which might not be 100% accurate. There were other decision‐influencing factors for women with ESBC to choose different surgery that was not addressed in this study. For example, individual beliefs for choosing mastectomy (avoiding radiation, less chance of reoccurrence), influencing choice for BCT (body image, minor surgery), family history of BC, and surgeon factors (individual surgeon practice, higher caseload).^21^

## 5 CONCLUSION

Distance to the nearest or the nearest accessible RTF influences the type of surgery received. Especially among women in Louisiana ≥65 years with ESBC who are eligible for BCT, the type of surgery received is associated with the closest RTF or the nearest one that accepts the woman's insurance. Future studies are encouraged to consider the accessibility of patients to radiation therapy and distance–age interaction to have precise results. Target intervention to help old women overcome the transportation barrier may increase BCT.

## AUTHOR CONTRIBUTIONS

**Pratibha Shrestha:** Conceptualization (lead); data curation (equal); formal analysis (lead); investigation (lead); methodology (lead); software (lead); writing – original draft (lead). **Quyen D. Chu:** Conceptualization (lead); writing – review and editing (equal). **Mei‐Chin Hsieh:** Writing – review and editing (equal). **Yong Yi:** Data curation (supporting); writing – review and editing (equal). **Edward S. Peters:** Writing – review and editing (equal). **Edward Trapido:** Writing – review and editing (equal). **Qingzhao Yu:** Writing – review and editing (equal). **Tekeda Ferguson:** Writing – review and editing (supporting). **Xiao‐Cheng Wu:** Conceptualization (equal); funding acquisition (lead); methodology (equal); project administration (lead); supervision (lead); validation (lead); writing – original draft (lead); writing – review and editing (lead).

## FUNDING INFORMATION

This study is supported by NCI‐SEER (HHSN261201800007I/HHSN26100002) and CDC‐NPCR (NU58DP006332).

## CONFLICT OF INTEREST

No conflict of interest to disclose.

## ETHICS STATEMENT

This study was approved and reviewed by Louisiana State University Health Science Center Institutional Review Board (IRB#2368).

## Data Availability

The datasets used for this study are available from the Louisiana Tumor Registry (LTR) after the LTR's Research Committee approves the data request proposal.
